# The individuals’ awareness and adoption of electronic health records in China: a questionnaire survey of 1,337 individuals

**DOI:** 10.1186/s12889-024-18423-y

**Published:** 2024-03-27

**Authors:** Yizhou Xu, Zongmin Pei, Xing He, Lu Guo, Li Zeng, Xiaoxuan Huang, Jian Zhang

**Affiliations:** 1 Department of Operations Management, Sichuan Provincial People’s Hospital, School of medicine, University of Electronic Science and Technology of China, Chengdu, China; 2 Department of Cardiology, The Second Affiliated Hospital of Chengdu University of Traditional Chinese Medicine, Chengdu, China; 3 Department of Psychosomatic Medicine, Chengdu Seventh people’s Hospital (Affiliated Cancer Hospital of Chengdu Medical College), Chengdu, China; 4 Department of Pulmonary and Critical Care Medicine, Sichuan Provincial People’s Hospital, School of Medicine, University of Electronic Science and Technology of China, Chengdu, China

**Keywords:** Electronic health record, Physical examination, Digitization

## Abstract

**Background:**

Electronic health records (EHRs) are digital records of individual health information. However, their adoption and utilization remain low. This study explores the factors influencing the implementation of EHRs through a questionnaire survey to enhance individual awareness and adoption of EHRs.

**Methods:**

A questionnaire and an expert rating scale were developed sequentially, and the consistency of the scores from five experts was calculated using Kendall’s W to generate a final questionnaire. A non-parametric test was utilized to analyze differences in continuous data that did not follow a normal distribution. Categorical variables were expressed as percentages (%), the chi-square test was employed for group comparisons, and multiple logistic regression was implemented to assess individuals’ awareness and adoption of EHRs.

**Results:**

In total, 1,341 survey questionnaires were distributed between January and December 2022, with 1,337 valid responses (99.7%). The results indicated that the proportion of participants who were aware of EHRs and had a bachelor’s degree or higher education, an income of ≥$700 per month, residence in urban areas, possessed self-care abilities, and underwent annual physical examinations was significantly higher than that without awareness of EHRs (*P* < 0.05), while in hearing problems and walking abilities was markedly lower than that of participants without awareness of EHRs (*P* < 0.05). Additionally, the proportion of individuals willing to self-manage EHRs was significantly higher than those reluctant to do so (*P* < 0.05) among participants with a bachelor’s degree or higher education, an income of ≥$700 per month, residence in urban areas, possession of self-care abilities, annual physical examinations, hearing problems, and poor walking abilities. Age (Odds Ratio [OR] = 1.104, 95% Confidence Interval [CI] 1.001–1.028, *P* = 0.033), hearing problems (OR = 0.604, 95% CI 0.377–0.967, *P* = 0.036), self-care ability (OR = 5.881, 95% CI 1.867–18.529, *P* = 0.002), and annual physical examinations (OR = 3.167, 95% CI 2.31–4.34, *P* < 0.001) were independently associated with willingness to self-manage EHRs. Annual physical examination (OR = 2.507, 95%CI 1.585–2.669, *P* < 0.001) also independently made a difference to the awareness of EHRs.

**Conclusions:**

Our findings suggest that annual physical examinations, age, hearing problems, and self-care abilities are significant factors in assessing individuals’ awareness and adoption of EHRs. Understanding the characteristics of individuals who are aware of or are willing to take advantage of EHRs plays a positive role in promoting their popularization and application.

**Supplementary Information:**

The online version contains supplementary material available at 10.1186/s12889-024-18423-y.

## Introduction

Electronic health records (EHRs) are digital archives generated by individuals to document health-related behaviors, which have value for preservation. They can capture data across all medical institutions and store them securely and confidentially in a computer system designed to serve individuals throughout their lifetime [1]. Digitally stored data can be shared across different medical institutions, enabling medical workers to exchange healthcare data and granting individuals’ access to quality medical services. A statistical analysis in 2018 demonstrated that electronic records accounted for 97.8% of health records among permanent resident populations in China, with a reporting rate of 75%. The reporting rate of primary health facilities reached 85%, whereas the actual utilization rate of EHRs was not accounted for [2]. Surveys in the United States, South Korea, Japan, and Australia revealed low rates of EHRs utilization [3, 4]. EHRs are a publicly funded priority in developed countries. Almost all medical institutions in the United States have adopted them since the 1970s. In recent decades, the primary practical use of EHRs in the United States has been for medical billing, while research, teaching, and health management have been secondary uses [5]. In the past five years, EHRs have often served as tools for public health surveillance. However, there has been insufficient investment in promoting public self-health management [6]. In most developing countries, including India, Sierra Leone, and Malawi, the adoption and implementation of EHRs are mainly limited to diseases, such as human immunodeficiency virus/acquired immunodeficiency syndrome and tuberculosis, in local areas of these countries. However, efforts are being made to integrate EHRs into national healthcare systems using e-health strategies. This integration aims to facilitate individual healthcare services, guide resource allocation and utilization, and promote data sharing and use across these countries [7].

To date, there has been a considerable amount of research on EHRs and encryption technology, clinical disease, and psychological problems. However, there remains an inadequate level of general awareness and attention towards the value of utilizing EHRs among individuals, governments, archiving fields, and medical fields. Some individuals are reluctant to cooperate with medical institutions to extract personal health data, and effective synergies have not been formed among various institutions, resulting in EHRs not being fully utilized [8, 9].

To promote the adoption of EHRs in the population, this study conducted a questionnaire survey among residents in Hainan and Chengdu, China, to explore the factors influencing awareness and self-management of EHRs.

## Methods

### Study subjects

In this prospective study, participants were recruited from Hainan and Chengdu, China, from January to December 2022. This study was approved by the Ethics Committee of the Sichuan Provincial People’s Hospital (No. LS (Y) 2022 − 360) and written informed consent was obtained from all participants.

The participants were required to meet the inclusion criteria of being 18 years of age or older, without intellectual disabilities, and capable of cooperating to complete the questionnaire. The participants were excluded if they refused to participate or were unable to complete the questionnaire.

### Questionnaire design

A questionnaire was initially compiled covering the individuals’ information, health status, and EHR-related information on a 4-point scale (1 = unimportant, 2 = less important, 3 = important, and 4 = more important). The expert inclusion criteria were: (1) senior professional title; (2) Doctor of Medicine; (3) five years or more research experience in the field of public health; and (4) voluntary participation in this study, possessing relevant knowledge, and providing valuable advice.

Afterwards, questionnaires with scores greater than three (on a 4-point scale) were retained, which were subsequently revised and supplemented according to 5 experts’ opinions, resulting in a final questionnaire with 15 questions.

### Data collection

The research was disseminated in these two ways: First is to go to large communities to conduct offline surveys with community residents. The other is to publicize through online promotion methods [10].

The survey data included demographic information such as age, gender (male/ female), education background (below or above the bachelor’s degree), income (<$700 per month/≥$700 per month), residence (suburban/ urban), household size (< 3 persons/≥3 persons), and presence of children, health-related information such as vision problems (visual impairments such as myopia, hyperopia, cataracts, and glaucoma / no vision impairment), hearing problems (with/without hearing dysfunction), walking abilities (with/without gait dysfunction), chronic diseases (presence/absence), self-care abilities (with/without self-care abilities), and annual physical examinations, as well as EHR-related information, including awareness of EHRs and willingness to self-manage EHRs. Using an empirical estimation method [11], it was estimated that at least 300 questionnaires would be collected by setting the sample size to 20 times the number of variables.

### Statistical analysis

Continuous data that did not conform to a normal distribution are expressed as medians (Q1-Q3), and nonparametric tests were performed to analyze the differences. Categorical variables were analyzed as percentages (%). Chi-square tests were used for group comparisons, Kendall’s W correlation test was conducted to evaluate the consistency of expert scores [12], and multivariate logistic regression analysis was performed to investigate factors related to participants’ awareness and self-management of EHRs. Statistical analyses were performed using SPSS version 26.0, with a *p*-value < 0.05 considered statistically significant.

### Flow chart

**Fig. 1:**
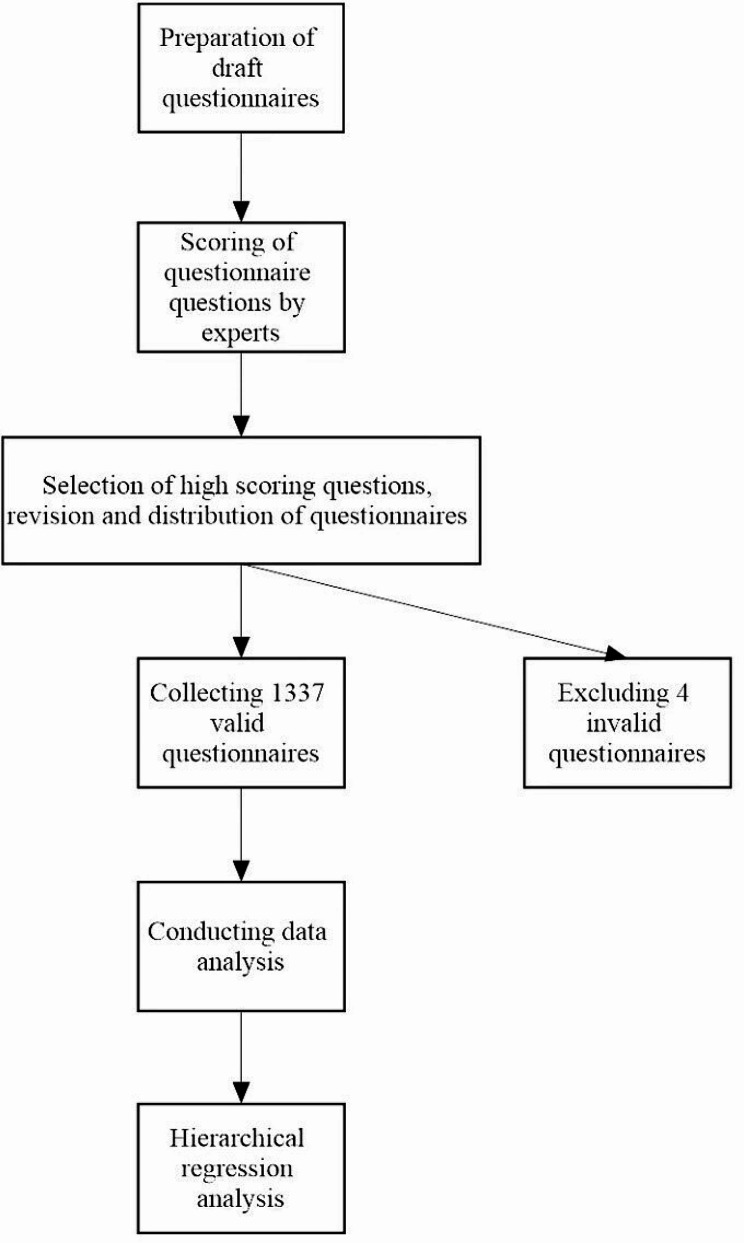
Flow chart of the questionnaire survey

The flow chart of the detailed operation is shown in Fig. 1.

## Results

### Expert participants information

Domain experts were invited to assess the questions and a correlation analysis of their opinions was conducted using the Delphi method. The results showed good consistency among the experts’ questionnaire scores (Kendall’s W = 0.471, *P* = 0.003; Table 1).

**Table 1:** Consistency of the consultation results

Experts(n)	Items(n)	Kendall’s W	χ^2^	*P*
5	15	0.471	32.958	0.003

### Basic information of the participants

In total, 1,341 survey questionnaires were distributed from January 2022 to December 2022, with 1,337 valid responses (99.7%). The results indicated that the median age of the participants was 38 years old, and 833 were female (62.3%); 1,101 (82.3%) participants had a bachelor’s degree or above, 798 (59.7%) had a monthly income ≥$700, 521 (39%) had visual impairment, 1,317 (98.5%) had self-care abilities, 959 (71.7%) underwent annual physical examinations, 723 (54.1%) were aware of EHRs, and 1,098 (82.1%) were willing to self-manage EHRs (Table 2).

**Table 2:** Demographic characteristics of participants

	Category	Median (Q1-Q3)/ percentages n (%)
Age	-	38(29–48)
Gender	Male	504(37.7)
	Female	833(62.3)
Education background	Below bachelor’s degree	236(17.7)
	Bachelor’s degree or above	1101(82.3)
Income	<$700 per month	539(40.3)
	≥$700 per month	798(59.7)
Residence	Suburban	331(24.8)
	Urban	1006(75.2)
Household size	<3 persons	454(34)
	≥ 3 persons	883(66)
Presence of children	No	409(30.6)
	Yes	928(69.4)
Vision problem	No	816(61)
	Yes	521(39)
Hearing problem	No	1192(89.2)
	Yes	145(10.8)
Walking ability	No	39(2.9)
	Yes	1298(97.1)
Chronic disease	No	994(74.3)
	Yes	343(25.7)
Self-care ability	No	20(1.5)
	Yes	1317(98.5)
Annual physical examination	No	378(28.3)
	Yes	959(71.7)
Awareness of EHRs	No	614(45.9)
	Yes	723(54.1)
Willingness to self-manage EHRs	No	239(17.9)
	Yes	1098(82.1)

### Participants’ awareness of EHRs

Based on their awareness of EHRs, the participants were divided into two groups: 723 (54.1%) with EHR awareness and 614 (45.9%) without. The proportion of participants aware of EHRs, who had a bachelor’s degree or above, an income of ≥$700 per month, resided in urban areas, possessed self-care abilities, and underwent annual physical examinations, was significantly higher than that without EHR awareness (*P* < 0.05), whereas the proportion with hearing problems and poor walking abilities was markedly lower than that of participants without EHR awareness (*P* < 0.05) (Table 3).

**Table 3:** The analysis of participants’ awareness toward EHRs

	Category	Awareness of EHRs		Z/χ^2^		*P*
		No	Yes			
Age		38(28–48)	39(29–48)		-1.038	0.299
Gender	Male	248(49.2)	256(50.8)		3.51	0.061
	Female	366(43.9)	467(56.1)			
Education background	Below bachelor’s degree	132(55.9)	104(44.1)		11.56	0.001^*^
	Bachelor’s degree or above	482(43.8)	619(56.2)			
Income	<$700 per month	275(51)	264(49)		9.446	0.002^*^
	≥$700 per month	339(42.5)	459(57.5)			
Residence	Suburban	173(52.3)	158(47.7)		7.125	0.008^*^
	Urban	441(43.8)	565(56.2)			
Household size	<3 persons	206(45.4)	248(54.6)		0.084	0.773
	≥ 3 persons	408(46.2)	475(53.8)			
Presence of children	No	199(48.7)	210(51.3)		1.77	0.183
	Yes	415(44.7)	513(55.3)			
Vision problem	No	239(45.9)	282(54.1)		0.001	0.976
	Yes	375(46.0)	441(54)			
Hearing problem	No	533(44.7)	659(55.3)		6.469	0.011^*^
	Yes	81(55.9)	64(44.1)			
Walking ability	No	588(45.3)	710(54.7)		6.96	0.008^*^
	Yes	26(66.7)	13(33.3)			
Chronic disease	No	444(44.7)	550(55.3)		2.46	0.117
	Yes	170(49.6)	173(50.4)			
Self-care ability	No	15(75)	5(25)		6.912	0.009^*^
	Yes	599(45.5)	718(54.5)			
Annual physical examination	No	229(60.6)	149(39.4)		45.596	< 0.001^*^
	Yes	385(40.1)	574(59.9)			

^*^P < 0.05

### Participants’ willingness to self-manage EHRs

It was reported that 1,098 participants (82.1%) were willing to self-manage their EHRs, whereas the remaining 239 (17.9%) were not. The proportion of participants willing to self-manage EHRs was observably higher among those who had a bachelor’s degree or above, an income of ≥$700 per month, residence in urban areas, possessed self-care abilities, underwent annual physical examinations, hearing problems, and poor walking abilities, compared to those unwilling to self-manage EHRs (*P* < 0.05) (Table 4).

**Table 4:** Participants’ willingness to self-manage EHRs

		Willingness to self-manage EHRs		Z/χ^2^		*P*
		No	Yes			
Age		35(27–46)	39(29–48)		-2.586	0.01^*^
Gender	Male	89(17.7)	415(82.3)		0.026	0.872
	Female	150(18)	683(82)			
Education background	Below bachelor’s degree	53(22.5)	183(77.5)		4.098	0.043^*^
	Bachelor’s degree or above	186(16.9)	915(83.1)			
Income	<$700 per month	123(22.8)	416(77.2)		15.037	< 0.001^*^
	≥$700 per month	116(14.5)	682(85.5)			
Residence	Suburban	89(26.9)	242(73.1)		24.339	< 0.001^*^
	Urban	150(14.9)	856(85.1)			
Household size	<3 persons	79(17.4)	375(82.6)		0.106	0.745
	≥ 3 persons	160(18.1)	723(81.9)			
Presence of children	No	83(20.3)	326(79.7)		2.346	0.126
	Yes	156(16.8)	772(83.2)			
Vision problem	No	84(16.1)	437(83.9)		1.787	0.181
	Yes	155(19)	661(81)			
Hearing problem	No	201(16.9)	991(83.1)		7.689	0.006^*^
	Yes	38(26.2)	107(73.8)			
Walking ability	No	225(17.3)	1073(82.7)		8.887	0.003^*^
	Yes	14(35.9)	25(64.1)			
Chronic disease	No	171(17.2)	823(82.8)		1.194	0.275
	Yes	68(19.8)	275(80.2)			
Self-care ability	No	12(60)	8(40)		24.541	< 0.001^*^
	Yes	227(17.2)	1090(82.8)			
Annual physical examination	No	125(33.1)	253(66.9)		82.861	< 0.001^*^
	Yes	114(11.9)	845(88.1)			

^*^P < 0.05

### Factors influencing the awareness of EHRs

Multivariate regression analysis was performed to assess the potential factors influencing the awareness of EHRs. The analysis revealed that undergoing annual physical examination (Odds Ratio [OR] = 2.507, 95% Confidence Interval [CI] 1.585–2.669, *P* < 0.001) was independently associated with participants’ awareness of EHRs (Table 5).

**Table 5:** Multivariate regression analysis of factors influencing the awareness of EHRs

	B	Standard error	Wald	OR	95%CI		*P*
Age(median age at 39)	0.005	0.005	0.983	1.005	0.995	1.014	0.321
Gender(Male)	0.178	0.117	2.289	1.194	0.949	1.503	0.13
Education background (Bachelor’s degree or above)	0.284	0.17	2.795	1.329	0.952	1.854	0.095
Income (≥$700 per month)	0.084	0.13	0.417	1.087	0.843	1.402	0.519
Residence(Urban)	-0.004	0.145	0.001	0.996	0.974	1.323	0.975
Hearing problem	-0.328	-0.195	2.837	0.721	0.492	1.055	0.092
Walking ability	-0.305	0.404	0.571	0.731	0.334	1.626	0.45
Self-care ability	0.801	0.586	1.87	2.229	0.707	7.03	0.171
Annual physical examination	0.721	0.133	29.483	2.507	1.585	2.669	< 0.001^*^

^*^P < 0.05

### Factors influencing the willingness to self-manage EHRs

A multivariate regression analysis was carried out to examine the factors influencing participants’ willingness to self-manage EHRs. The results uncovered that age (OR = 1.104, 95% CI 1.001–1.028, *P* = 0.033), hearing problems (OR = 0.604, 95% CI 0.377–0.967, *P* = 0.036), self-care abilities (OR = 5.881, 95% CI 1.867–18.529, *P* = 0.002) and undergoing annual physical examinations (OR = 3.167, 95% CI 2.31–4.34, *P* < 0.001) were independently correlative factors affecting participants’ willingness to self-manage EHRs (Table 6).

**Table 6:** Multivariate regression analysis of factors influencing the willingness to self-manage EHRs

	B	Standard error	Wald	OR	95%CI		*P*
Age(median age at 39)	0.014	0.007	4.553	1.014	1.001	1.028	0.033^*^
Gender(Male)	-0.066	0.157	0.174	0.936	0.688	1.275	0.667
Education background (Bachelor’s degree or above)	-0.007	0.214	0.001	0.993	0.653	1.511	0.975
Income (≥$700 per month)	0.083	0.17	0.237	1.086	0.778	1.517	0.627
Residence (Urban)	0.29	0.18	2.612	1.337	0.94	1.902	0.106
Hearing impairment	-0.505	0.241	4.407	0.604	0.377	0.967	0.036^*^
Poor walking ability	0.009	0.484	< 0.001	1.009	0.391	2.605	0.985
Self-care ability	1.77	0.586	9.157	5.881	1.867	18.529	0.002^*^
Annual physical examination	1.153	0.161	51.347	3.167	2.31	4.34	< 0.001^*^

^*^P < 0.05

## Discussion

EHRs play a crucial role in both medical support and chronic disease management [13], as they can support public health care, facilitating tracking of personal medical history, and aid in detecting general health problems. In this study, the factors influencing the awareness and adoption of EHRs were monitored using a questionnaire survey, which is of great value for boosting the popularization and application of EHRs in the general population.

During the formulation of the questionnaire, the Delphi method for expert evaluation was applied, and the outcomes reflected average-to-good consistency, signifying that the questionnaire had a certain investigative value that could mirror the participants’ actual situation. The results of the data acquisition revealed that just over half of the recruited participants conveyed knowledge about EHRs, suggesting considerable room for improving the popularity of EHRs. Furthermore, most participants were willing to self-manage their EHRs, indicating a high degree of health concern.

Our study confirmed that the proportion of participants with awareness of EHRs who had a bachelor’s degree or above, a monthly income of ≥$700, resided in urban areas, underwent annual physical examinations, and possessed walking and self-care abilities was significantly higher than those without awareness of EHRs. However, the proportion with hearing impairment and poor walking abilities was notably lower than that of participants without awareness of EHRs. That is because the two major factors, hearing impairment and poor walking abilities, may limit their ability to receive information and pose greater challenges in social communication compared to individuals without such limitations, leading to a lack of awareness about EHRs among most of them [14, 15].

The proportion of participants willing to self-manage EHRs, who had an average age of 39 years, a bachelor’s degree or above, an income of ≥$700 per month, resided in urban areas, possessed self-care abilities, underwent annual physical examinations, experienced hearing problems, and had walking abilities, was significantly higher than that of those who were unwilling to self-manage EHRs. A survey conducted on the degree of EHRs adoption in Austria demonstrated that the complexity of individuals’ health problems correlated positively with the degree of EHRs adoption, and older age groups paid more attention to EHRs [16]. In our study, hearing problems, walking abilities, and self-care abilities mirrored the complexity of individual health problems, which are considered important factors affecting individuals’ awareness of and willingness to utilize EHRs.

According to a survey conducted among individuals using EHRs in several countries, including Sweden and Finland [17, 18], the majority reported experiencing varying degrees of chronic diseases, suggesting that individuals with different degrees of physical problems focused more on their own health. Our results revealed that higher-income individuals had a significantly higher awareness of and concern about their own health. A study conducted in 2023 found that low- and middle-income countries lagged behind in the adoption and implementation of EHRs, with significant potential for improvement [19]. The proportion of participants aware of EHRs was significantly higher among those with a bachelor’s degree or above, indicating that educational background had an effect on the participants’ attention to EHRs and consequently led to disparities in self-health awareness [20]. This underscores considerable potential to improve the popularity of EHRs among low-income and low-education individuals.

The number of urban residents who know and focus on EHRs is increasing, which is believed to be related to the distribution and popularization of medical resources in China. Medical resources are significantly more abundant in urban areas than in suburban areas [21, 22]; therefore, urban residents have access to more convenient and high-quality medical resources and services [23]. Therefore, their awareness of and emphasis on healthcare were also significantly higher than those of suburban residents.

The results of the regression analysis showed that annual physical examinations were an independent influencing factor for awareness and self-management of EHRs, and individuals who undergo annual physical examinations were more aware of EHRs and willing to self-manage them. They not only have more opportunities to learn about EHR-related information [24], contributing to significant differences in their awareness of EHRs, but are also more concerned about their own health, which is a common trait across countries [25, 26]. Therefore, they are more willing to manage their EHRs, creating a virtuous cycle. In addition, age, hearing problems, and self-care abilities independently affect EHRs self-management. Older individuals and those with self-care abilities are more inclined to self-manage EHRs, while those with hearing problems are reluctant to do so. Individuals with limited self-care abilities require assistance from others to perform activities of daily living; therefore, those with self-care abilities are better at self-management. Moreover, age is a risk factor for many diseases [27], and with increasing age, individuals cannot help but turn their focus to physical health instead of other aspects [28]. This highlights the purpose and significance of EHRs in assisting individuals with long-term records and follow-up of their health status, facilitating improvements in health and medical care. Individuals with hearing impairment are reluctant to self-manage ERHs due to the challenges they face in social interactions and daily life, which contribute to a lack of awareness regarding self-management. The discomfort caused by hearing problems may make EHRs more of a burden than a tool for help [29], which reminds us to pay more attention to these individuals when promoting EHRs.

When promoting and popularizing EHRs, we can consider linking hospitals with communities, increasing the connection between clinical staff and the general population, and strengthening our understanding of EHRs [30]. Online medical services can complement the use of EHRs by offering features such as the provision of online family doctors. This convenience helps to improve the adoption of EHRs and increases the willingness of the public to use them.

### Limitations

This study had some limitations. First, Likert-type questionnaire options were not adopted in the questionnaire design, rendering quantitative analysis through dimension division impractical. Second, the study was limited to the Chengdu and Hainan in China, excluding individuals from other regions. This limitation could affect the results due to regional factors such as economy, healthcare, and education. Finally, this study did not incorporate the practical use of EHRs or related factors within the population, besides, the occupations of the population were not surveyed.

## Conclusion

In summary, our findings demonstrate that annual physical examinations are a crucial factor affecting individuals’ awareness and adoption of EHRs. Additionally, older age, hearing problems, and self-care abilities were important factors influencing individuals’ willingness to use EHRs, suggesting that strengthening the publicity of EHRs for these individuals can better promote the practical application of EHRs. Our study provides important information on EHRs in health management and disease control.

### Electronic supplementary material

Below is the link to the electronic supplementary material.

Supplementary Material 1

## Data Availability

Data availability statement: All relevant data are within the manuscript.

## References

[1] Callahan A, Shah NH, Chen JH. Ann Intern Med. 2020;172(11 Suppl):S79–84. Research and Reporting Considerations for Observational Studies Using Electronic Health Record Data[J]. 10.7326/M19-0873 PMC7413106 32479175

[2] Liang J, Li Y, Zhang Z (2021). Adoption of Electronic Health Records (EHRs) in China during the past 10 years: consecutive Survey Data Analysis and comparison of sino-american challenges and Experiences[J]. J Med Internet Res.

[3] Kruse CS, Stein A, Thomas H (2018). The use of Electronic Health Records To Support Population Health: a systematic review of the Literature[J]. J Med Syst.

[4] Tapuria A, Porat T, Kalra D (2021). Impact of patient access to their electronic health record: systematic review[J]. Inf Health Soc Care.

[5] Kim E, Rubinstein SM, Nead KT (2019). The Evolving Use of Electronic Health Records (EHR) for Research[J]. Semin Radiat Oncol.

[6] Knicely K, Loonsk JW, Hamilton JJ et al. Electronic case reporting development, implementation, and expansion in the United States[J]. Public Health Rep, 2024:2081019800. 10.1177/00333549241227160 PMC11284980 38411134

[7] Kumar M, Mostafa J (2019). Research evidence on strategies enabling integration of electronic health records in the health care systems of low- and middle-income countries: a literature review[J]. Int J Health Plann Manage.

[8] Xia Z, Gao W, Wei X et al. Perceived Value of Electronic Medical Records in Community Health Services: a National Cross-sectional Survey of Primary Care workers in Mainland China[J]. Int J Environ Res Public Health, 2020,17(22). 10.3390/ijerph17228510 PMC7698410 33212868

[9] Lin H, Tang X, Shen P (2018). Using big data to improve cardiovascular care and outcomes in China: a protocol for the CHinese electronic health Records Research in Yinzhou (CHERRY) Study[J]. BMJ Open.

[10] Berndt AE (2020). Sampling Methods[J]. J Hum Lact.

[11] Norman G, Monteiro S, Salama S (2012). Sample size calculations: should the emperor’s clothes be off the peg or made to measure?[J]. BMJ.

[12] Premelc J, Vuckovic G, James N (2019). Reliability of judging in DanceSport[J]. Front Psychol.

[13] Zanaboni P, Kummervold PE, Sorensen T (2020). Patient Use and Experience with Online Access to Electronic Health Records in Norway: results from an online Survey[J]. J Med Internet Res.

[14] Carter C, Boisvert I, Docking K (2023). Communication, academic and social outcomes of childhood cancer survivors with hearing loss: a systematic review[J]. Pediatr Blood Cancer.

[15] Wang J, Kwan P, Zhang G (2023). A Multidimensional Assessment of activities of Daily Living, Mental Status, Communication, and Social abilities among older adults in Shenzhen, China: cross-sectional Study[J]. JMIR Public Health Surveill.

[16] Halmdienst N, Pruckner GJ, Winter-Ebmer R (2023). Complexities of health and acceptance of electronic health records for the Austrian elderly population[J]. Eur J Health Econ.

[17] Kujala S, Horhammer I, Vayrynen A (2022). Patients’ experiences of web-based Access to Electronic Health Records in Finland: cross-sectional Survey[J]. J Med Internet Res.

[18] Moll J, Rexhepi H, Cajander A (2018). Patients’ experiences of accessing their Electronic Health records: National Patient Survey in Sweden[J]. J Med Internet Res.

[19] Ye J, Xiong S, Wang T (2023). The Roles of Electronic Health Records for Clinical Trials in low- and Middle-Income countries: scoping Review[J]. JMIR Med Inf.

[20] Chen L, Hong J, Xiong D (2020). Are parents’ education levels associated with either their oral health knowledge or their children’s oral health behaviors? A survey of 8446 families in Wuhan[J]. BMC Oral Health.

[21] Chen Y, Yin Z, Xie Q (2014). Suggestions to ameliorate the inequity in urban/rural allocation of healthcare resources in China[J]. Int J Equity Health.

[22] Yi M, Peng J, Zhang L (2020). Is the allocation of medical and health resources effective? Characteristic facts from regional heterogeneity in China[J]. Int J Equity Health.

[23] Jiang Y, Cai X, Wang Y et al. Assessment of the supply/demand balance of medical resources in Beijing from the perspective of hierarchical diagnosis and treatment[J]. Geospat Health, 2023,18(2). 10.4081/gh.2023.1228 37831418

[24] Kim YJ, Park H. Big Data. 2019;7(3):163–75. Improving Prediction of High-Cost Health Care Users with Medical Check-Up Data[J]. 10.1089/big.2018.0096 31246499

[25] Hills O, Shah D. Online health information seeking, medical care beliefs and timeliness of medical check-ups among African Americans[J]. Patient Educ Couns; 2020. 10.1016/j.pec.2020.06.006 32616321

[26] Kherad O, Carneiro AV (2023). General health check-ups: to check or not to check? A question of choosing wisely[J]. Eur J Intern Med.

[27] Vinik AI, Camacho P, Reddy S (2017). AGING, DIABETES, AND FALLS[J]. Endocr Pract.

[28] Nelson NA, Jacobucci R, Grimm KJ (2020). The bidirectional relationship between physical health and memory[J]. Psychol Aging.

[29] Trott M, Smith L, Xiao T (2021). Hearing impairment and diverse health outcomes: an umbrella review of meta-analyses of observational studies[J]. Wien Klin Wochenschr.

[30] Nguyen L, Bellucci E, Nguyen LT (2014). Electronic health records implementation: an evaluation of information system impact and contingency factors[J]. Int J Med Inf.

