# Supplementary material for: The individuals’ awareness and adoption of electronic health records in China: a questionnaire survey of 1,337 individuals
Source: BMC Public Health. 2024 Mar 27;24:905. doi: 10.1186/s12889-024-18423-y (PMC10967175; doi:10.1186/s12889-024-18423-y)
Supplement: Supplementary file 1 — Supplementary Material 1 [file 12889_2024_18423_MOESM1_ESM.docx]

**Questionnaire on Individuals' Needs for Electronic Health Records**

Dear Friends:

In response to the call of " Be the first line of defense in own health" and to promote the concept of active ageing and successful ageing, we are conducting a questionnaire survey on the "Demand for Personal Electronic Health Record". Personal electronic health record is an electronic record of personal health information, including basic personal information, summary of major diseases and health problems, and medical and health service records.

We would like to invite you to participate in this study which has been reviewed and approved by the Medical Ethical Committee of Sichuan Provincial People's Hospital, University of Electronic Science and Technology of China. Please read the following carefully before deciding whether to participate in this study or not. Participating in this study will not involve any cost issues or adverse reactions. This study aims to use blockchain technology to build a new type of trustworthy digital health record platform and provide cross-regional exemplary applications, and the necessary information that you will need to provide us with or allow us to collect includes: your personal information (gender, income, etc.), your health condition, whether willing to self-manage EHRs or not，etc. In the process of completing this questionnaire, if some questions in the questionnaire make you feel discomfort, you can end the questionnaire at any time. You may refuse to take part or withdraw at any time at any stage of completing the questionnaire and this will not affect you in any way, so please do not have any concerns.

In order to ensure the accuracy of the survey results, this questionnaire is anonymous, and we will keep the contents of the questionnaire completely confidential, and all information will be used for academic research only.

**1.** **What’s your gender? ( )**

| A. Male |
| --- |
| B. Female |

**2. How old are you? ________**

**3. What’s your education background? ( )**

| A. Below bachelor’s degree |
| --- |
| B. Bachelor’s degree or above |

**4.What is your average monthly income right now（more than 5,000 RMB per month or below）? ( )**

| A. <$700 per month |
| --- |
| B. ≥$700 per month |

**5. Where do you currently live in（in the urban or in the suburb）？**

| A. Suburban |
| --- |
| B. Urban |

**6. How many people live together in your household？**

| A.＜3 persons |
| --- |
| B.≥3 persons |

**7. Do you have any children？**

| A. No |
| --- |
| B. Yes |

**8. Do you have any vision problems (visual impairments such as myopia, hyperopia, cataracts, glaucoma, etc) ？（ ）**

| A. No |
| --- |
| B. Yes |

**9. Do you have** **hearing problems（hearing problems such as hearing loss、auditory hallucination, etc）? ( )**

| A. No |
| --- |
| B. Yes |

**10. Is there any problems with your walking ability(walking problems such as lameness, paralysis of the lower limbs, walking slowness, etc)? （ ）**

| A. No |
| --- |
| B. Yes |

**11. Do you have any chronic diseases（chronic diseases such as hypertension, diabetes, and coronary heart disease, etc）? ( )**

| A. No |
| --- |
| B. Yes |

**12. Do you have self-care ability(self-care ability means that you can conduct your daily life without the help of others)? ( )**

| A. No |
| --- |
| B. Yes |

**13. Whether you will have an annual physical examination or not? ( )**

| A. No |
| --- |
| B. Yes |

**14.Do you know what electronic health records are?**

| A. No |
| --- |
| B. Yes |

**15. Would you like to manage your own electronic health records by yourself?**

| A. No |
| --- |
| B. Yes |

**Your questionnaire ends here, thank you for taking the time.**

**Hope everything goes your way.**
